# Collaboration strategies affecting implementation of a cross-systems intervention for child welfare and substance use treatment: a mixed methods analysis

**DOI:** 10.1186/s43058-024-00666-w

**Published:** 2024-11-11

**Authors:** Emmeline Chuang, Alicia Bunger, Rebecca Smith, Amanda Girth, Rebecca Phillips, Edward Miech, Kathryn Lancaster, Jared Martin, Fawn Gadel, Marla Himmeger, Jen McClellan, Jennifer Millisor, Tina Willauer, Byron J. Powell, Elinam Dellor, Gregory A. Aarons

**Affiliations:** 1 University of California, Berkeley, Berkeley, CA USA; 2 Ohio State University, Columbus, OH USA; 3 Western Oregon University, Monmouth, OR USA; 4 Indiana University School of Medicine, Bloomington, IN USA; 5 Wake Forest University, Winston-Salem, NC USA; 6 University of California, San Francisco, CA USA; 7 Public Children Services Association of Ohio, Columbus, OH USA; 8 Children and Family Futures, Irvine, CA USA; 9 Washington University in St. Louis, St. Louis, MO USA; 10 University of California San Diego, San Diego, CA USA

**Keywords:** Cross-system interventions, Coincidence analysis, Collaboration, Fidelity

## Abstract

**Background:**

Collaboration strategies refer to policies and practices used to align operations and services across organizations or systems. These strategies can influence implementation of cross-system interventions focused on improving integration of care, but remain under-specified and under-examined. This study identifies collaboration strategies and the conditions under which they affected implementation of Sobriety Treatment and Recovery Teams (START), an evidence-based intervention focused on integrating child welfare and behavioral health services for families involved with both systems.

**Methods:**

Our study sample included 17 county child welfare agencies that implemented START. Data on collaboration strategies and organizational context were obtained from key informant interviews, frontline worker surveys, and contracts. Contextual data were drawn from secondary data, and fidelity data were drawn from an administrative database. Qualitative and quantitative data were integrated using coincidence analysis, and used to identify combinations of conditions that uniquely differentiated agencies with higher and lower fidelity to START.

**Results:**

Fidelity was lower for intervention components requiring cross-system collaboration. Although key informants acknowledged the importance of collaboration for START implementation, few agencies used formal collaboration strategies other than staff co-location or reported high communication quality between frontline staff in child welfare and behavioral health. In coincidence analysis, four conditions differentiated agencies with higher and lower fidelity with 100% consistency and 88% coverage. We found that either strong leadership support or, in high need communities, third-party resource support from local behavioral health boards were sufficient for high fidelity. Similarly, in high need communities, absence of third-party resource support was sufficient for low fidelity, while in low need communities, absence of communication quality was sufficient for low fidelity.

**Conclusion:**

Administrators, frontline workers, and interested third parties (i.e., other stakeholders not directly involved in implementation) can use collaboration strategies to facilitate implementation. However, the effectiveness of collaboration strategies depends on local context. In agencies where internal leadership support for implementation is low but need for intervention is high, third-party resource support may still be sufficient for high fidelity. Further research is needed to test effectiveness of collaboration strategies in different conditions and on a broader range of process and implementation outcomes.

**Trials registration:**

ClinicalTrials.gov, NCT03931005, Registered 04/29/2019, https://classic.clinicaltrials.gov/ct2/show/NCT03931005.

**Supplementary Information:**

The online version contains supplementary material available at 10.1186/s43058-024-00666-w.

Contributions to the literature • This study advances our understanding of how collaboration strategies can be used by administrators, frontline workers, and interested third parties not directly involved in implementation to facilitate implementation of evidence-based, cross-system interventions.

• Organizations could benefit from more systematic use of formal collaboration strategies to improve communication quality among front-line staff.

• It is important to understand how local contextual factors can impact effectiveness of collaboration strategies.

• In communities with high service need, third parties not directly involved in an intervention can still help support implementation even when local leadership support is low.

## Background

Cross-system interventions that integrate services or activities across different organizations or sectors of care can improve client outcomes and enhance community impact [1–3]. However, these interventions can be difficult to implement and sustain due to the need to coordinate activities across stakeholders that may have differing missions, professional roles, capacity, or modes for distributing resources [4, 5]. Collaboration strategies refer to policies and practices used to align operations and services across organizations or systems in the implementation of evidence-based, cross-system interventions [6]. These strategies can take different forms (e.g., data sharing agreements, joint service or staffing arrangements), and may be deployed by different stakeholders, from frontline staff to organizational leaders or third-party brokers.

Despite the importance of strong collaboration for successful implementation and sustainment of evidence-based, cross-system interventions [7, 8], little is known about the use and impact of collaboration strategies in implementation or whether certain strategies may be more effective than others. The current study seeks to address this gap by examining collaboration strategies and the conditions under which they impacted the implementation of an evidence-based intervention, Sobriety Treatment and Recovery Teams (START). START is a cross-system intervention focused on integrating child welfare and behavioral health systems for families involved with child welfare due to parental substance use [9, 10].

### Importance of cross-system collaboration for families involved with child welfare due to parental substance use

In 2022, U.S. child welfare agencies received more than 4.2 million referrals to investigate alleged maltreatment of more than 7.5 million children [11]. Child maltreatment is a serious public health concern. Children who have experienced maltreatment are at increased risk for a number of adverse developmental, health, and mental health outcomes, including learning problems, problems relating to peers, depression, and higher risk of engaging in high-risk or negative health behaviors in adolescence and adulthood [12–14].

Alcohol and/or drug misuse are among the most prevalent caregiver risk factors for maltreatment [15], particularly for children three years or younger [16]. Caregiver substance use disorder (SUD) has also been associated with increased complexity and severity of child maltreatment [17, 18]. SUD treatment can support improved outcomes for families, particularly if services are initiated soon after initial involvement with the child welfare system and coordinated with child welfare services [19–21]. However, many caregivers struggle to engage in treatment within child welfare permanency timelines mandated by the 1997 United States Adoption and Safe Families Act [22].

Limited child welfare caseworker knowledge of substance use disorders or treatment resources can delay referrals to treatment [23, 24]. Following referral, limited availability of treatment programs that address ancillary service needs of caregivers involved with the child welfare system can delay access to treatment [25–27]. These challenges are often further exacerbated by differing priorities, perspectives, and information-sharing processes of the child welfare, court, and substance use treatment systems with which families are involved [28–30].

Effective cross-systems collaboration between child welfare and substance use treatment systems can improve caregiver access and engagement in treatment and increase likelihood of reunification [31–33], but is challenging to achieve, particularly in the absence of institutionalized, multilevel supports for collaboration [5, 28]. Identifying effective collaboration strategies is essential for successful implementation of cross-system interventions. However, cross-system collaboration strategies remain under-specified, and limited information exists regarding their effectiveness under different conditions.

### Sobriety Treatment and Recovery Teams (START)

**Fig. 1:**
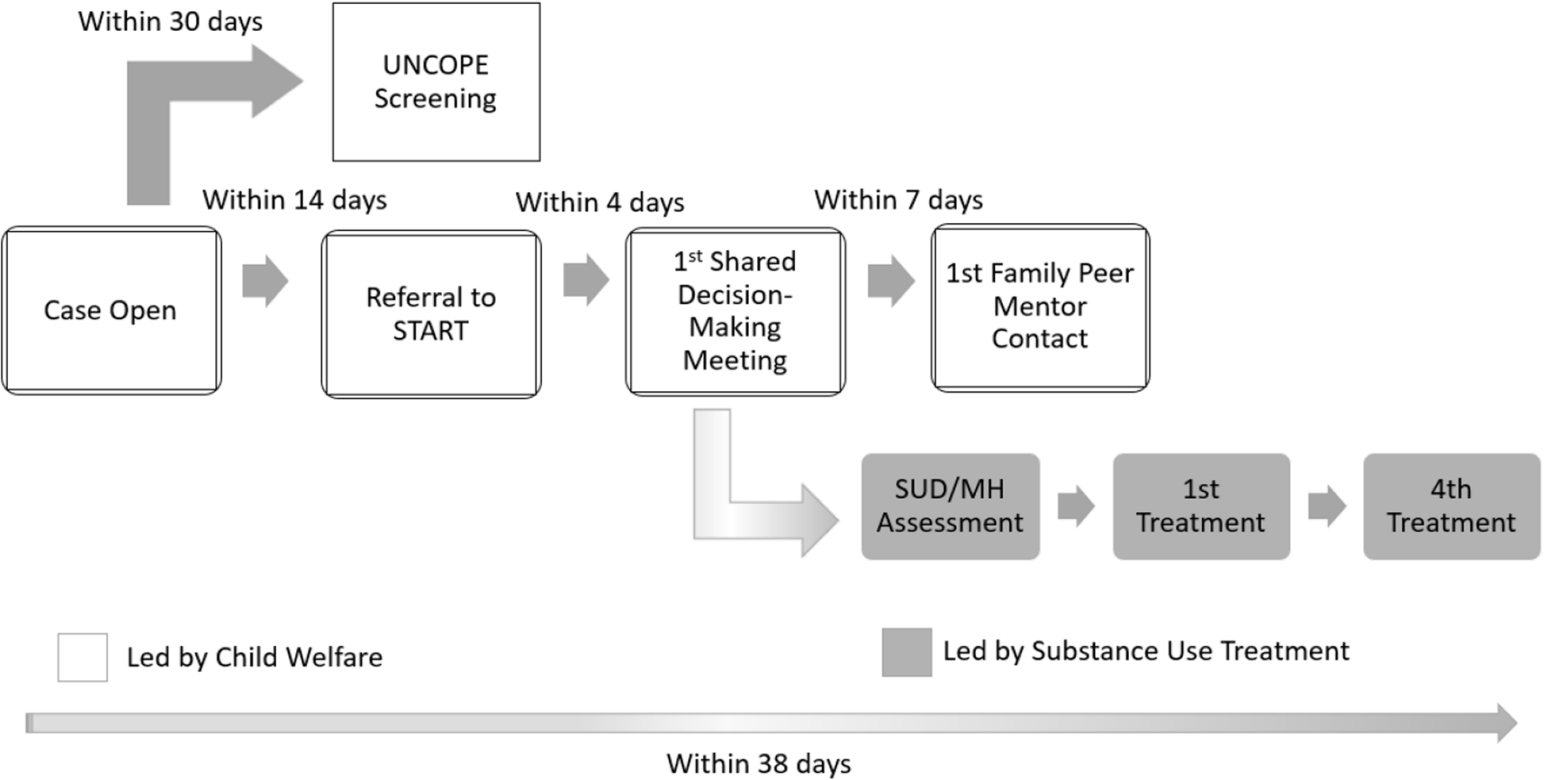
Sobriety Treatment and Recovery Teams (START) timeline. UNCOPE is a validated, brief screening instrument for substance abuse treatment (Proctor & Hoffmann, 2016). SUD/MH = Substance use and mental health. SUD screening is recommended but not required by the national START model, and was voluntarily adopted by Ohio START

START is an evidence-based, cross-systems intervention for families with co-occurring child maltreatment and substance use. The intervention includes several sequenced practice components delivered across child welfare agency and substance use treatment systems, including screening caregivers for substance use disorders, shared decision-making meetings to plan services, wraparound support from a family peer mentor with lived experience in recovery and child welfare, and referral to a behavioral health treatment provider for assessment and at least four treatment sessions within 38 days of entering the child welfare system. A brief overview of key components of the model is provided in Fig. 1. When implemented as intended, START can expedite caregiver access and engagement in substance use treatment, increase likelihood of family reunification, and reduce subsequent maltreatment risk [9, 10, 34, 35]. However, successful implementation is contingent on a variety of contextual and organizational factors, including quality of collaboration between the child welfare and behavioral health systems [36].

### Conceptual model

**Fig. 2:**
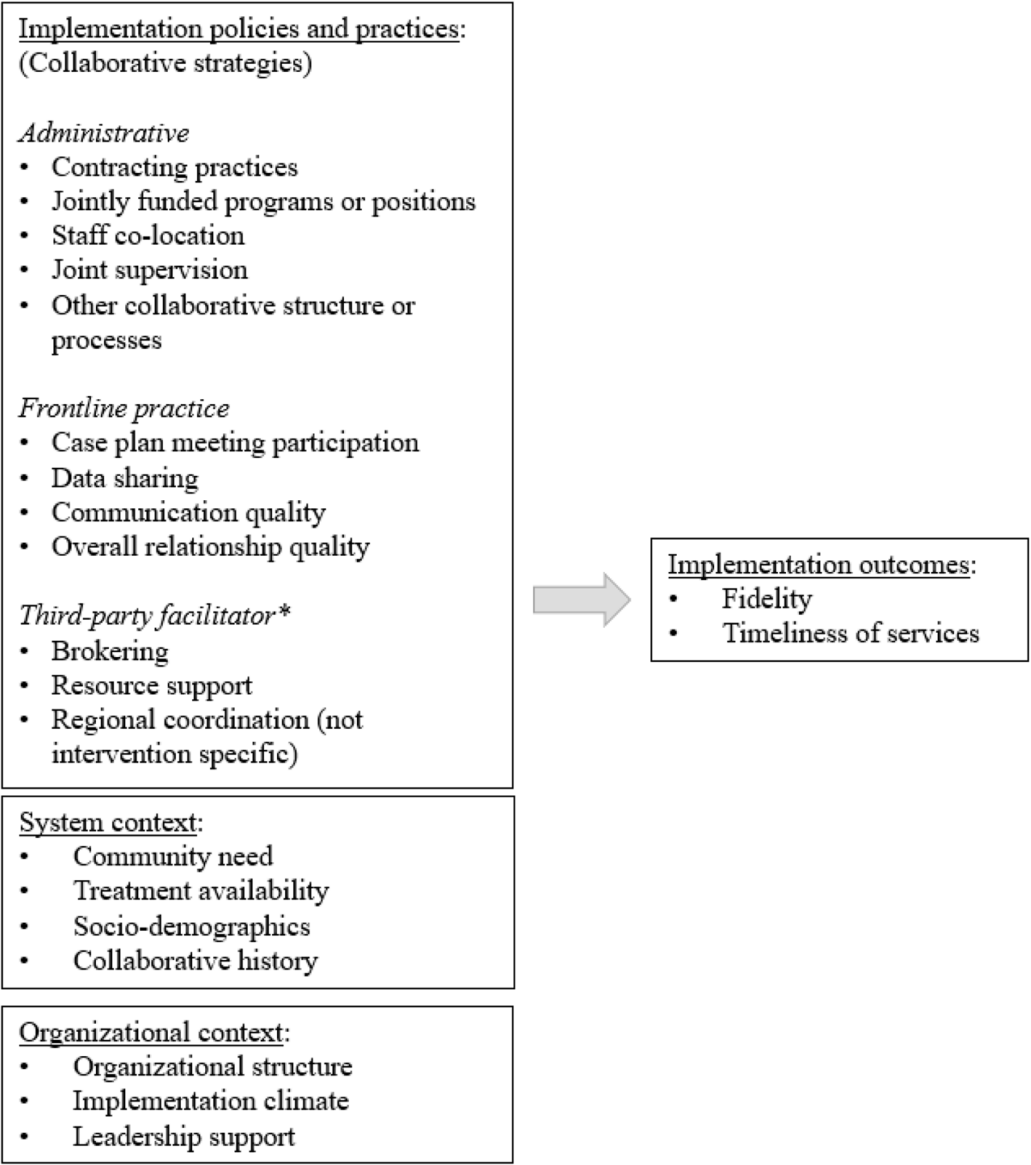
Conceptual framework of factors influencing START implementation outcomes. * In Ohio, local behavioral health boards are responsible for coordinating publicly funded behavioral health prevention, treatment, and recovery supports and could thus also help facilitate child welfare and substance abuse treatment system alignment efforts

Our study of collaboration strategies influencing START implementation is anchored by a conceptual model informed by the cross-sector alignment framework [37] and the Exploration, Preparation, Implementation, and Sustainment (EPIS) framework (see Fig. 2 and Appendix 1 for more information). In our model, collaboration strategies are defined as implementation policies and practices used to align operations and services across organizations [38]. These strategies can take multiple forms, may be implemented by different stakeholders (e.g., administrators, frontline staff, third parties), and their effectiveness may depend on the local system and organizational context in which START is being implemented.

Given the growing number of evidence-based interventions with cross-systems service integration components, there is a need to better understand which collaboration strategies are most effective at improving systems alignment in different contexts. Informed by this conceptual model, we draw on data from the Ohio START initiative to identify collaboration strategies and other contextual and organizational factors associated with intervention fidelity. Fidelity refers to the extent to which an intervention is implemented as intended by the intervention developer [39], and strongly influences whether evidence-based interventions yield expected outcomes when implemented in new settings [40, 41].

## Methods

### Study design

We used a convergent mixed methods approach, in which qualitative and quantitative data from the Ohio START evaluation were independently collected and then integrated during analyses [42]. First, we collected and analyzed data from qualitative key informant interviews and child welfare agency contracts with substance use treatment providers to identify collaboration strategies used to facilitate START implementation. We also descriptively analyzed administrative data, frontline worker surveys and other secondary data to obtain information on fidelity and on other contextual and organizational factors hypothesized to influence fidelity. We then integrated these data in analyses using a configurational comparative method known as coincidence analysis [43]. This study is reported using the Good Reporting of a Mixed Methods Study (GRAMMS) checklist [42].

### Study context and sample

In 2017, Ohio began implementing START under the leadership of the Public Children Services Association of Ohio (PCSAO), a nonprofit organization that represents Ohio’s 85 county-based public child welfare agencies. In Ohio, child welfare services are state supervised but county administered, meaning individual counties have considerable discretion in how services are operated and delivered; thus, our unit of analysis was the county child welfare agency. Because START implementation was staggered, the current study draws on data from 17 of 30 child welfare agencies in the first two cohorts that implemented START (Cohort 1 *n* = 9; Cohort 2 *n* = 8). These 17 agencies were purposively selected to maximize variation in local system context (e.g., geographic diversity, agency funding, and governance structure), and agreed to participate in a mixed-methods evaluation of START implementation. Most agencies (*n* = 11; 64.7%) were in rural counties, 2 were suburban, and 4 were urban; 6 were in the Appalachian region.

### Data sources

Data used in this study include key informant interviews, child welfare agency contracts with substance use treatment providers, frontline worker surveys, secondary data on local system context, and administrative data on START fidelity.

*Key informant interviews.* Between January 2020 and April 2021, we conducted 48 small group interviews with 104 unique participants involved in START implementation with the 17 child welfare agencies. This included 17 interviews with 52 child welfare staff (leaders, supervisors, and frontline workers), 25 interviews with 44 behavioral health treatment providers, and 6 interviews with behavioral health board representatives responsible for planning, evaluating, funding, and coordinating local mental health and substance use treatment services. Additional information about interview procedures is available in Appendix 2 and elsewhere [38].

With respondents’ permission, all interviews were recorded and professionally transcribed; team members also completed debrief summaries after each interview to highlight key themes and guide initial codebook development. All transcripts were coded by two investigators in the qualitative software NVivo 12.0 using template analysis, in which an initial codebook reflecting key content domains of interest was refined to incorporate emergent themes [44]. Any discrepancies were discussed until consensus was reached. Coded data were reviewed with a 16-member expert panel to specify and validate collaboration strategies; additional information about this process is available elsewhere [6]. Data were subsequently re-analyzed to determine the presence or absence of collaboration strategies within each agency and determine perceived quality of collaboration between child welfare and behavioral health.

*Child welfare agency contracts with substance use treatment providers.* We collected all child welfare agency contracts executed with behavioral health providers and pertaining to START between 2018 and 2020. When properly specified and enforced, contracts can support inter-agency collaboration by providing clarity and direction for resource allocation, accountability, shared decision-making, and coordination [45, 46]. In the current study, START-related contracts in each agency were reviewed and coded to obtain information on presence and type of formal agreement between child welfare agencies and behavioral health providers, provider roles and responsibilities, performance expectations, data sharing expectations, and specificity of contract terms.

*Frontline worker surveys.* We surveyed all child welfare caseworkers serving START families in fall 2020, approximately 2–3 years after initial START adoption. Surveys were conducted as part of the larger, statewide evaluation of START and assessed behavioral health referral patterns, quality of collaboration, implementation climate, and implementation leadership. Questions about behavioral health referral patterns were used to identify behavioral health partners and the average % of cases referred for services within county as opposed to outside of county. Collaboration quality was assessed using the Wilder Collaboration Factors Inventory, which is comprised of 47 items across six categories of factors shown to affect the success of collaboration endeavors. Implementation climate was assessed using the 6-item Implementation Climate Measure [47], and implementation leadership was assessed using the 12-item Implementation Leadership Scale [48]. Survey questions were reviewed by community partners and internally pilot tested prior to administration.

Surveys were distributed to a total of 174 workers in the 17 participating agencies. Survey participation was voluntary, and informed consent was obtained prior to administering surveys. A total of 140 workers completed the survey (between 3 and 34 per agency; overall response rate 80.5%). Survey measures were aggregated to the child welfare agency level to calculate a mean score for each participating agency.

*Secondary data on local system context*. Data reflecting local system context were drawn from multiple sources, including 2018 data on county child welfare agency structure and statistics from the Ohio Department of Job and Family Services, behavioral health board structure from the Ohio Association of County Behavioral Health Authorities, opioid-related overdose deaths from the Ohio Department of Health, naloxone administration rates from the Ohio Department of Public Safety, suspected drug overdoses among emergency department (ED) visits of Ohio residents aged 11 years and older from the Ohio Department of Health Violence and Injury Prevention, rates of neonatal abstinence syndrome (NAS) among resident live births from the Ohio Hospital Association and the Ohio Public Health Information Warehouse, numbers of children in child welfare custody (per 1000), screened in child maltreatment reports, and cases opened from the PCSAO Factbook, and county sociodemographic characteristics from the U.S. Census Bureau.

*Fidelity data.* Fidelity data were collected using the Needs Portal, a web-based management information system used by child welfare caseworkers and participating providers to track and share client-level information in real time, e.g., about assessments, service referrals, treatment modalities, treatment service dates, or other contacts with clients [49, 50]. As part of standard case reporting procedures, START team members prospectively entered information about whether and when each component of START was delivered. In the current study, data from March 2019 to October 2022 were cleaned and aggregated to the child welfare agency level to differentiate agencies with higher vs. lower START fidelity.

### Outcome measure

Our dependent variable was fidelity, or the degree to which START was implemented as intended. Consistent with Carroll et al. (2007)’s fidelity framework [39], our measure of fidelity focused on adherence to core elements of the START model. In Ohio, START is comprised of multiple, sequenced components expected to occur within specified time periods (see Fig. 1), including (a) completion of a brief, substance use screen (UNCOPE) within 30 days of the child welfare case opening [51]; (b) first shared decision-making meeting (SDMM), in which child welfare, behavioral health, other service professionals, and families come together to plan services, within 4 days of referral to START; (c) first contact with a family peer mentor within 7 days of the SDMM; (d) comprehensive behavioral health assessment within four days of the SDMM; (e) first substance use treatment session within four days of assessment; and (f) completion of four substance use treatment sessions within 12 days of assessment. These components were identified as critical for fidelity in prior studies of the START model [9] and were also prioritized by our community partners and by START developers in assessing adherence to START.

In measuring fidelity, we focused on coverage based on number of model components delivered and duration based on whether the components were delivered in a timely way. We first used data from the Needs Portal to examine the percentage of cases that received each intervention component, the timeliness of service receipt, and variation across all county child welfare agencies in the state (see Table 1). Agencies were considered high fidelity if they were above statewide averages in the percentage of cases receiving each intervention component and in timeliness with which services were received. Based on feedback from our state partner (PCSAO), we revised our fidelity measure to omit component (f) (completion of four substance use treatment sessions within 12 days of assessment); this fidelity indicator was perceived as less within child welfare or START partnership control and as needing to be modeled separately.

**Table 1:** Ohio START Fidelity

	% Cases receiving			% Cases timely (out of those that received the component)		
	Statewide average	Agency minimum	Agency maximum	Statewide average	Agency minimum	Agency maximum
Substance use disorder screening	89%	0%	100%	93%	58%	100%
Shared decision-making meeting	89%	56%	100%	62%	0%	100%
Family peer mentor contact	70%	0%	100%	45%	0%	100%
Comprehensive behavioral health assessment	62%	0%	100%	57%	0%	100%
Any substance use treatment^a^	58%	0%	100%	55%	0%	100%
At least four substance use treatment sessions	47%	0%	88%	43%	0%	100%

^a^% Cases timely for this component is calculated as the number of cases that received first treatment visit within 4 days of assessment / number of cases with any substance abuse treatment visit logged

### Coincidence analysis

In the analytic phase, a configurational comparative method (CCM) known as coincidence analysis was used to integrate our qualitative and quantitative data to identify collaboration strategies and other factors that differentiated agencies with high vs. low START fidelity. Additional information about CCMs is provided in Appendix 4.

In applying CNA, we first separately analyzed data from key informant interviews, child welfare agency contracts, frontline worker surveys, and secondary data to develop an initial list of 61 potential explanatory factors (see Appendix 3); of these, 28 were measures of collaboration strategies identified in Fig. 2 and the remaining measures reflected organizational and contextual factors hypothesized in our conceptual model as influencing fidelity. All explanatory factors were calibrated for use in CNA, meaning they were recoded as binary (yes/no) or categorical variables (max value range 1–5). Data were then entered into an Excel spreadsheet, in which rows represented each agency and columns reflected specific variables of interest.

The next step was to reduce the number of conditions in our data. We did this by applying the “minimally sufficient conditions” (“msc”) function within the R coincidence analysis (“cna”) package [43] to identify configurations of conditions with particularly strong connections to high or low fidelity. We considered all one-, two-, and three-condition configurations that met pre-designated thresholds for consistency and coverage. Consistency indicates how often a solution results in the outcome when present, and coverage indicates empirical prevalence of the solution within the sample (i.e., number of cases with configuration and outcome present divided by the total number of cases with the outcome). We started with a consistency threshold of 75%, and assessed all configurations that satisfied coverage thresholds of 75%, 80%, 85%, 90%, and 95% to compare output at different thresholds.

We generated a condition table listing all configurations of conditions that met these thresholds. Review of this table resulted in identification of smaller subsets of conditions to model. During this phase, we also used mathematical output from the -msc- function and subject matter knowledge to further reduced the dimensionality of selected factors, e.g., dichotomizing certain variables rather than preserving their original, categorical format. Models were developed iteratively using appropriate functions within the R “cna” package, based on our assessment of overall consistency and coverage of identified solutions and potential model ambiguity (i.e., when competing models explain the outcome equally well based on their consistency and coverage scores). We prioritized configurations with high consistency, lower complexity (i.e., fewer conditions), higher coverage scores (i.e., % cases for which the outcome is explained by the solution), and for which different values of the same conditions explained both high and low fidelity. We also assessed models for potential model ambiguity (when competing models explain the outcome equally well, based on consistency and coverage scores) [52].

To help mitigate risk of overfitting the data (i.e., including causally irrelevant factors), final model selection was based on fit-robustness (FR) scores, followed by highest consistency, highest coverage, and lowest complexity. Table 2 provides a description of conditions included in our final coincidence analysis model. All analyses were conducted using the R “cna” package, R Studio, and Excel.

**Table 2:** Explanatory conditions included in final coincidence analysis model

Condition	Description	Data source
***Collaboration strategy – Third party broker***		
Resource support	Whether the behavioral health board provided any funding or resources for the START program or its clients (1 = Yes, 0 = No)	Key informant interviews
***Collaboration strategy – Frontline practice***		
High communication quality	Perceived quality of cross-agency communication, measured using salient 5-item subscale from the Wilder Collaboration Factors Inventory; responses aggregated to agency level (1 = High if agency-level response in top tercile; else = 0)	Frontline worker survey
***Organizational context***		
High leadership support	Perceived leadership support for START implementation, measured using 12-item Implementation Leadership Scale; responses aggregated to agency level (1 = High if agency-level response in top tercile; else = 0)	Frontline worker survey
***System context – community need***		
High community need	Whether agency was in the top quartile within the state for total number of reports screened in for investigation, the number of cases transferred for ongoing case management, and the number of children in child welfare custody (1 = Yes, 0 = No)	Ohio Department of Job and Family Services

## Results

### Fidelity

We rated each county child welfare agency in our sample using our measure of fidelity. Of the 17 agencies in our study sample, 6 agencies were classified as high fidelity, i.e., above statewide average in the percentage of cases receiving each intervention component and the timeliness with which services were received; the remaining 11 agencies were classified as low fidelity.

### Collaboration strategies

Participating agencies varied in type and usage of different collaboration strategies for aligning child welfare and behavioral health providers for START implementation (Table 3). At the administrative level, the most commonly used collaboration strategy was to co-locate a family peer mentor employed by a behavioral health provider with child welfare caseworkers (71%; 12 of 17) and the least commonly used strategy was to jointly fund a program or position (0%). Most agencies (59%) chose to jointly supervise family peer mentors with behavioral health provider partners, and most (59%) also had START-specific contracts in place; however, few agencies (11%) had high-quality contracts in place (i.e., contracts with clear goals and performance metrics). In qualitative interviews, participants varied in their perceptions of contracts, with some describing the contracts as valuable for setting clear expectations and providing role clarity and others perceiving them as unnecessary “*as long as they talk to us*,* as long as there is communication*.”

**Table 3:** Collaboration strategies used in START implementation (*n* = 17)^a^

Strategy	Definition	Frequency (%)
***Administrative***		
Contracting practices: Any use of contract	Whether agency has a START-specific contract in place (yes/no)	59
	Whether a START contract was signed before first START family served	47
Contracting practices: High contract quality	High contract quality based on presence of clear goals and performance metrics	11
Contracting practices: High contract specificity	Contract specifies method and frequency with which START-specific data or other information will be shared	35
Jointly funded programs or staff	Contract allows for jointly funded program or positions between child welfare and behavioral health	0
Staff co-location	Family peer mentor role contracted out to behavioral health but co-located with child welfare	71
Joint supervision	Family peer mentor co-supervised by behavioral health and child welfare	59
***Frontline practice***		
Regular case plan meeting participation	Regular behavioral health provider participates in shared decision-making meetings	35
Consistent data sharing	Child welfare staff and behavioral health providers consistently exchange needed information about clients and services	65
High communication quality	High quality cross-agency communication (5-item subscale of the WCFI^a^)	24
High overall relationship quality	Mutual child welfare and behavioral health satisfaction with the child welfare-behavioral health relationship	41
***Third party facilitator***		
Regional coordination	Regional behavioral health board member(s) part of county START steering committee	53
	Other formal mechanisms in place within the county for facilitating cross-sector collaboration (e.g., coalition, drug court)	53
Brokering	Regional behavioral health board helped connect child welfare agencies with behavioral health providers for START	41
Resource support	Regional behavioral health board provided any funding for START program or START clients	24

^a^Appendix 3 provides a full list of collaboration strategies assessed, their definition, and our initial approach to calibration. A dichotomized subset of these strategies (15 of 28) is presented here for illustrative purposes only

At the frontline level, use of formal strategies for promoting system alignment was relatively rare, with most child welfare caseworkers and behavioral health providers relying primarily on informal relationships and information exchange. For example, while child welfare caseworkers and behavioral health providers in most agencies (65%; 11 of 17) reported consistent exchange of needed information about clients and services, only 35% did so via formally established channels or procedures, and only 35% reported regular behavioral health provider participation in SDMM. In qualitative interviews, participants indicated that lack of time and/or resources to pay for therapists’ time were barriers to behavioral health therapist participation in SDMM. As one child welfare agency leader noted, “*Even when we tried conference call or scheduling it [SDMM] at clients’ typically scheduled session time*,* it wasn’t well received [by therapists]… because it’s not a Medicaid billable hour… or at least not billable at a clinical or case management rate.”*

Analyses of aggregated frontline worker survey data revealed that the quality of communication between child welfare and behavioral health was rated highly in less than a quarter (24%; 4 of 17) of agencies. While many participants reported improvements in communication following START implementation, particularly at the administrative level, there were only four agencies in which child welfare caseworkers, family peer mentors, and behavioral health providers all reported high quality, bidirectional communication at the frontlines of care. As a behavioral health director working with one of these agencies noted, *“We have a great relationship with Children’s Services… We are there to serve their clients*,* they’re there to help us do that*,* communication is excellent*,* whatever I need from them they’re usually very responsive and same back to them… We have weekly updates on every client that we serve.”* Participants from most agencies were more likely to identify gaps in communication and ways in which they wished communication could be improved. As another behavioral health supervisor working with a different agency noted, *“I wish there was a better working relationship… versus working opposed to each other… A lot of our clinicians and therapists hear one side of things from clients and feel like their clients aren’t being treated fairly and when they have reached out to advocate*,* they’re not heard because phone calls don’t get returned… We have tried to provide brief reports to Children’s Services to help them [but] struggled with them doing the same thing*,* letting us know what’s going on.”*

When asked about other stakeholder engagement systems alignment efforts, most agencies (71%; 12 of 17) reported at least some engagement from behavioral health boards in START, though less than a quarter (24%; 4 of 17) described regular, START-specific engagement. Instead, most agencies described behavioral health boards as either not involved or engaged only sporadically or “as needed.” Key informants in over half of agencies (53%; 9 of 17) also described presence of other formal mechanisms for facilitating collaboration between child welfare and behavioral health providers in their communities, such as community coalitions or a family dependency treatment court.

When asked about specific strategies used to facilitate system alignment, key informants most frequently described inclusion of behavioral health board representatives on START steering committees (53%; 9 of 17), assistance from boards in identifying high-quality behavioral health providers in the community to contract with for services (41%; 7 of 17), or direct provision of resources to support START program implementation or fund treatment for clients (24%; 4 of 17). In qualitative interviews, participants noted that behavioral health board engagement in START varied depending on factors such as the number of counties a board oversaw and board members’ prior relationship with child welfare. Board engagement was higher in communities where child welfare agency leaders had previously collaborated with board members through joint participation in other community coalitions, advisory councils, or initiatives. As one highly engaged behavioral health board director noted, *“We have a lot of history working with child welfare [in this county] on a wraparound program through the Family & Children First Council [local collaborative for issues affecting children and families]… where our board*,* our local development disabilities board*,* and our child welfare board all contribute money to pooled funds to help families with multisystem high acuity needs*,* and that relationship goes back almost 30 years.”* This level of engagement was a sharp contrast to the much more limited board engagement reported by other child welfare agencies, some of whom were unaware of their local behavioral health board members were or how to leverage boards to benefit START implementation in their communities: *“We’ve had zero contact [with behavioral health board]. We haven’t reached out and neither have they… I don’t even know who the board members are*,* at least not in the last several years…”*.

### Coincidence analysis results

Using coincidence analysis, we identified several conditions whose presence or absence differentiated agencies with high vs. low fidelity (Table 4). Specifically, agencies with high START fidelity either had [1] high leadership support for START *or* [2] high community need for child welfare services and third party resource support from the behavioral health board. These conditions explained high fidelity in 5 of 6 agencies (83% coverage) with 100% consistency. Only one agency met criteria for both solution pathways (i.e., high leadership support *and* high community need for child welfare resources and resource support from the behavioral health board).

**Table 4:** Coincidence analysis results: factors that differentiate agencies with higher and lower START implementation

	High fidelity (*n* = 6)		Low fidelity (*n* = 11)	
	SP 1	SP 2	SP 3	SP 4
Third party resource support		●		○
High communication quality			○	
High leadership support	●			○
High community need		●	○	●
Number of agencies in SP^a^	3	3	6	4
Overall Model Consistency	100%		100%	
Overall Model Coverage	83%		91%	

*SP* Solution pathway

● = Presence, ○ = Absence

^a^1 high fidelity agency met conditions for both solution pathways

By contrast, agencies with low START fidelity either had (1) low communication quality and low community need *or* (2) high community need but low leadership support and no resource support from the behavioral health board. These conditions explained low fidelity in 10 of 11 agencies (91% coverage) with 100% consistency. There was no overlap between agencies in these two solution pathways.

## Discussion

Strong collaboration is critical for implementing interventions that integrate services across different organizations or systems [7, 8, 53]. However, there is limited research on collaboration strategies and the conditions under which these strategies are most effective at improving implementation. Consistent with prior research [1, 54], our study of Ohio START implementation confirmed the difficulty of maintaining fidelity when cross-sector collaboration is involved. In Ohio START, fidelity was higher for intervention components directly under child welfare agency control (e.g., brief SUD screens and SDMMs) and lower for components requiring collaboration with behavioral health providers (e.g., comprehensive behavioral health assessment and substance use treatment). These findings reinforce the importance of collaboration strategies, i.e., implementation policies and practices focused on aligning services or operations across disparate organizations or systems, for helping to strengthen linkages between inner and outer contexts when implementing cross-systems interventions [55].

In prior research, we identified seven different collaboration strategies used by stakeholders in implementing Ohio START [6]. In the current study, we found that while key informants in all participating agencies acknowledged the importance of cross-system collaboration for effective implementation, actual use of collaboration strategies for aligning child welfare and behavioral health was low, i.e., used by fewer than half of agencies. The most frequently used administrative strategy, co-location of peer mentors with child welfare caseworkers, was also the only one explicitly required as an implementation strategy by the national purveyor of the START model. At the frontlines, use of structured collaboration strategies such as a regular inclusion of behavioral health providers in SDMMs was relatively uncommon, with most frontline workers reporting reliance on informal relationships for sharing information. In qualitative interviews, we learned that behavioral health providers often did not participate in SDMMs because time spent in these meetings was not reimbursed, suggesting the need to carefully consider partner resource needs when developing budgets for implementation of cross-system interventions. Engagement of behavioral health boards as a third-party facilitator of collaboration (e.g., in brokering relationships or providing resources needed to support coordination efforts) was also not widespread, often due to limited county child welfare leadership awareness of board members or how they could be engaged to improve START implementation.

Perhaps because of the lack of formal supports for cross-sector collaboration, relationship quality and communication quality between child welfare and behavioral health was also relatively low in our sample. Evidence from other studies demonstrates how combining collaboration strategies at multiple levels (e.g., system, organizational, frontlines) of the system is important for effective service delivery [56–58]; the limited use of collaboration strategies might explain the fidelity gaps we observed in this analysis and suggests a need for supporting the development of strong multi-level collaborations for implementing Ohio START and other cross-system interventions.

### Collaboration strategies affecting fidelity

Coincidence analysis results provided broader insight into specific collaboration strategies and factors in the local community and organizational context that differentiated agencies with high vs. low fidelity. Specifically, the presence or absence of four conditions collectively explained fidelity in the majority of agencies in our sample: resource support from a third-party broker (county or multi-county behavioral health board), communication quality, leadership support, and severity of community need for child welfare services.

Consistent with prior literature on the importance of implementation leadership [59], we found that high child welfare leadership support for START was sufficient for high fidelity, regardless of whether an agency used any formal, collaboration strategies for bridging child welfare and substance use treatment systems. In communities where need for child welfare services was high, we found that third-party resource support from local behavioral health boards was also sufficient for high fidelity even when child welfare leadership support for START was low. In these communities, resource support from local behavioral health boards (key stakeholders in the “outer context”) could have served as an important substitute for “inner context” leadership in driving implementation. This finding is consistent with prior research demonstrating that leadership in both outer and inner contexts can influence evidence-based practice implementation and sustainment [59, 60]. Further research is needed to better understand how community dynamics and collaboration strategies used to bridge disparate contexts may interact with outer and inner context leadership support to influence implementation.

We also found that while presence of high quality communication was not by itself sufficient for high fidelity, the *absence* of high quality communication explained low fidelity in communities where need for child welfare services was lower. These findings reinforce prior research suggesting that high quality communication between frontline staff may be necessary but not sufficient for fidelity in the absence of other determinants of implementation [61].

### Limitations

Several limitations must be taken into consideration in interpreting study results. First, while our study included rich qualitative and quantitative data on a broad array of collaboration strategies and factors that could have influenced START fidelity, we did not include any data from clients. Client perspectives on extent to which services were client-centered, integrated, or responsive to their needs could provide important insights and should be addressed in future research. We also did not assess quality of intervention delivery, a moderator of fidelity that should be addressed in future research.

In addition, while coverage of the final solution model was high, two agencies were not included in any of the identified solution pathways, indicating a possible role for additional factors beyond those included in the model. Review of available data on these two agencies (1 high fidelity agency and 1 low fidelity agency) reveal that these agencies were similar in three of the four conditions included in the final solution model (no resource support from the board, low leadership support, high perceived quality of frontline communication), but differed in community context. Specifically, the high fidelity agency was located in a large, urban community in which need for child welfare services was high (51-74th percentile) but not in the highest quartile in the state, while the low fidelity agency was located in a smaller, Appalachian county where need for child welfare services was in the lowest quartile (< 25th percentile) in the state. Future research involving more nuanced measures of community context could explore in more depth the potential role of context on implementation outcomes.

Finally, our analyses examined whether collaboration strategies differentiated agencies with high vs. low fidelity, but did not assess their impact on perceived leadership support, relationship quality, communication quality, or other determinants of implementation. For example, it is possible that the impacts of some collaboration strategies on fidelity (e.g., data sharing agreements, staff co-location) were fully mediated by their impact on perceived leadership support or communication quality, two conditions that did emerge as meaningful in our analyses. Future research could more directly assess impact of different collaboration strategies on different determinants of implementation, rather than on more distal implementation outcomes.

### Implications for research, policy, and practice

Despite the limitations noted above, our study is the first to present and empirically test a conceptual model of different collaboration strategies hypothesized to affect implementation of evidence-based, cross-system interventions. Key study strengths include the inclusion of multilevel collaboration strategies, the collection and use of mixed methods data, and the application of an innovative method (coincidence analysis) to integrate these data in analyses. Study findings confirm that collaboration strategies are important for intervention fidelity and highlight the importance of considering equifinality in implementation research. To further refine and specify our conceptual model, additional research on collaboration strategies and implementation outcomes in different contexts is needed.

Our study also provides preliminary insights into how policymakers and practitioners can improve uptake of identified collaboration strategies. One particularly notable finding in our study was that local leaders often did not use identified collaboration strategies – particularly administrative and third-party facilitator strategies – simply because they were not aware of these strategies or how they might benefit START implementation. To address this gap, our study team used findings to develop a practitioner-oriented collaboration strategies toolkit, which we validated and distributed to community partners and Ohio START technical assistance providers. Alternative, evidence-informed approaches for raising awareness of collaboration strategies could include creating learning collaboratives or other opportunities for implementers from different agencies and community contexts to share strategies for addressing shared community needs and learn from one another. Future research could also empirically test effectiveness of these approaches at improving uptake of different collaboration strategies.

## Conclusions

Cross-system interventions, particularly those that integrate health, behavioral health, and social services, show promise for improving client outcomes and enhancing community impact if barriers to inter-agency collaboration can be overcome. This study identified four conditions that differentiated agencies with high vs. low START fidelity. Our findings emphasize the importance of collaboration strategies and of local community and organizational context to implementation of complex, cross-system interventions; in particular, findings suggest that while leadership support matters for implementation, resource support from interested third parties not directly involved in implementation can improve intervention fidelity in communities where need for intervention is high but internal leadership support is low.

## Supplementary Information

Supplementary Material 1.

## Data Availability

De-identified data will be made available from the first author upon reasonable request.

## Abbreviations

SDMM: Structured decision-making meetings
START: Sobriety Treatment and Recovery Teams
SUD: Substance use disorder
UNCOPE: Used, Neglected, Cut Down, Objected Preoccupied, Emotional Discomfort
