# Supplementary material for: Collaboration strategies affecting implementation of a cross-systems intervention for child welfare and substance use treatment: a mixed methods analysis
Source: Implement Sci Commun. 2024 Nov 11;5:127. doi: 10.1186/s43058-024-00666-w (PMC11552168; doi:10.1186/s43058-024-00666-w)
Supplement: Supplementary file 1 — Supplementary Material 1. [file 43058_2024_666_MOESM1_ESM.docx]

**Appendix 1. Conceptual framework and collaboration strategies**

Our study of collaboration strategies influencing START implementation is anchored by a conceptual model informed by the cross-sector alignment framework and the Exploration, Preparation, Implementation, and Sustainment (EPIS) framework. Briefly, the cross-sector alignment framework highlights the importance of partnership-level factors such as shared purpose, data, financing, and governance to system alignment efforts and helped inform our conceptualization of collaboration strategies. The EPIS framework describes implementation processes and contextual determinants of implementation including “bridging factors” and dynamic linkages between inner and outer contexts (40-42) that informed our selection of contextual and organizational factors hypothesized to influence implementation.

In our model, collaboration strategies are defined as implementation policies and practices used to align operations and services across organizations (44). These strategies can take multiple forms and may be implemented by different stakeholders (e.g., administrators, frontline staff, third parties). For example, at the administrative level, collaboration strategies could entail development of formal or informal agreements between child welfare agencies and behavioral health providers to contract for services, share data, or co-locate or cross-train staff. At the frontline, these strategies may reflect specific actions taken by practitioners to align operations or services for families (e.g., communication, data sharing, case plan meeting participation, etc.). Finally, collaboration strategies can also be enacted by third parties such as technical assistance providers or regional coordinating bodies (e.g., a multi-county behavioral health board), who facilitate collaboration between child welfare agencies and behavioral health providers by helping expedite or strengthen relationships, or by providing information or other resources needed to support cross- systems alignment (45, 46).

The effectiveness of these collaboration strategies may depend on the local system and organizational context in which START is being implemented. System context includes factors in the external environment such as community needs, treatment availability, and prior history of collaboration between different stakeholders in the community (40). Organizational context refers to factors within each participating organization that can also influence implementation, such as leadership support and readiness for change (40-42). As the number of evidence-based interventions with cross-systems service integration components continues to grow, there is a need to better understand which collaboration strategies are most effective at improving systems alignment in different contexts. Informed by this conceptual model, we draw on data from the Ohio START initiative to identify collaboration strategies and other contextual and organizational factors associated with intervention fidelity. Fidelity refers to the extent to which an intervention is implemented as intended by the intervention developer (43), and strongly influences whether evidence-based interventions yield expected outcomes when implemented in new settings (44, 45).

**Appendix 2. Key informant interview procedures**

Data sources used in this study include key informant interviews, child welfare agency contracts with substance use treatment providers, frontline worker surveys, secondary data on local system context, and administrative data on START fidelity. This appendix provides additional insight into key informant interview procedures.

***Key informant interviews***

Between January 2020 and April 2021, we conducted 48 small group interviews with 104 unique participants involved in START implementation with the 17 child welfare agencies. This included 17 interviews with 52 child welfare staff (leaders, supervisors, and frontline workers, including family peer mentors), 25 interviews with 44 behavioral health treatment providers (supervisors and clinicians), and 6 interviews with behavioral health board representatives. In Ohio, the public behavioral health system is comprised of county or multi-county behavioral health boards which are responsible for planning, evaluating, funding, and coordinating local mental health and substance use treatment services; we interviewed board representatives due to their potential role in facilitating inter-agency collaboration between local child welfare agencies and substance use treatment providers. To recruit interview participants, we began by sending an initial recruitment email to the main contact at each child welfare agency. We invited these contacts to participate in a small group interview and asked them to invite other child welfare agency staff involved in START implementation. To ensure inclusion of participants who could provide insight into different types of collaboration strategies, we worked closely with our contacts to recruit team members working at different hierarchical levels of the agency. During our interviews with child welfare agency team members, we asked them to identify their primary behavioral health partners. We then sent separate recruitment emails to behavioral health partners, who were interviewed separately. To obtain more information on collaboration strategies involving third-party facilitators, we also emailed recruitment invitations to behavioral health board directors. Prior to the interviews, all participants received the consent script and questions. Consent was obtained verbally at the beginning of each interview.

Interviews were on average 60 minutes long and conducted via video conference (Zoom) by at least two members of the research team, using a semi-structured interview guide. The interview guide addressed topics such as the status of START implementation, collaboration strategies used, perceived quality of collaboration between child welfare and behavioral health providers, relationships with other key stakeholders in the community (e.g., courts, behavioral health boards), and facilitators, barriers, and lessons learned in implementing START. For interviews conducted after March 2020, we also asked about pandemic impacts on collaboration and START implementation. Our interview guide was guided by our study aims and conceptual model, refined based on feedback from community partners in the child welfare and behavioral health systems, and internally pilot tested. Interview participants were offered a $30 gift card as a token of appreciation for their time.

**Appendix 3. Initial list of measures used in coincidence analysis (n=61)**

| **Measure** | | **Description** | **Values** | **Data Source** |  |  |
| --- | --- | --- | --- | --- | --- | --- |
| **Collaborative strategies** | **Administrative** | | | | | |
|  | Contracting practices | Child welfare agency has formal agreements in place with behavioral health partners | 0=0 partners, 1=1 partner, 2=2 partners, 3=3 partners, 4=4+ partners | Interview and contracts | |  |
|  |  | Percentage of behavioral health partners with which the child welfare agency has a formal agreement in place | 0 – 100% | Interview and contracts | |  |
|  |  | Whether county has a START contract in place | 0=No, 1=START language but not START-specific, 2= START-specific | Contracts | |  |
|  |  | Whether a START contract was signed before first START family served | 0=No, 1=Yes | Contracts and Needs Portal | |  |
|  |  | Contract quality (average across all contracts in county), assessed based on presence of clear goals and performance metrics (PM) | 0=No contract, 1=Low (no goals, no PM), 2=Moderate (goals or PM), 3=High (goals and PM) | Contracts | |  |
|  |  | Contract for START family peer mentor | 0=No, 1=Yes | Contracts | |  |
|  |  | Contract references “collaboration” or “partnership” | 0=Not present, 1= <3 times, 2=4-8 times, 3=9+ times | Contracts | |  |
|  |  | Contract specifies method and frequency with which data or other information will be shared between child welfare and behavioral health | 0=Not present; 1=Specifies method or frequency but not both; 2=Specifies both; 3=Specifies both and START-specific | Contracts | |  |
|  | Jointly funded programs or staff | Contract allows for jointly funded program or positions between child welfare and behavioral health | 0=No, 1=Yes | Contracts | |  |
|  | Staff co-location | Family peer mentor role contracted out to behavioral health but co-located with child welfare | 0=No, 1=Yes | Interviews | |  |
|  | Joint supervision | Family peer mentor co-supervised by behavioral health and child welfare | 0=No, 1=Intent to co-supervise but not fully implemented, 2=Co-supervision | Interviews | |  |
|  | Other collaborative structure or processes | Perceived quality of collaborative processes and structure (13-item subscale of the WCFI^a^) | 0=Below average, 1=Average, 2=Above average^b^ | FLW survey | |  |
|  | **Frontline** | | | | | |
|  | Case plan meeting participation | Behavioral health provider participates in shared decision-making meetings | 0=None, 1=Sporadic, 2=Regular/consistent participation | Interviews | |  |
|  | Data sharing | Child welfare staff and behavioral health providers exchange needed information about clients, services, etc. | 0=Low/limited, 1=Consistent but via informal processes only, 2=Consistent and formal | Interviews | |  |
|  | Communication quality | Perceived quality of interpersonal communication and working relationships between child welfare caseworker, family peer mentor, and behavioral health provider | 0=Low, 1=Moderate, 2=High | Interviews | |  |
|  |  | Perceived quality of cross-agency communication (5-item subscale of the WCFI^a^) | 0=Below average, 1=Average, 2=Above average^b^ | FLW survey | |  |
|  | Shared purpose | Clarity of collaborative purpose (7-item subscale of the WCFI^a^) | 0=Below average, 1=Average, 2=Above average^b^ | FLW survey | |  |
|  | Overall relationship quality | Child welfare satisfaction with their relationship with local behavioral health providers | 0=Low satisfaction, 1=Mixed, 2=High | Interviews | |  |
|  |  | Behavioral health provider satisfaction with their relationship with local child welfare agencies | 0=Low satisfaction, 1=Mixed, 2=High | Interviews | |  |
|  |  | Mutual satisfaction with the child welfare-behavioral health relationship | 0=No, 1=Yes | Interviews | |  |
|  |  | Overall quality of collaboration, full Wilder Collaboration Factors Inventory | 0=Below average, 1=Average, 2=Above average^b^ | FLW survey | |  |
|  | **Third party facilitator** | | | | | |
|  | Relationship with regional behavioral health board | Any regional behavioral health board engagement with START | 0=No, 1=Yes | Interviews | |  |
|  |  | Contract includes regional behavioral health board | 0=No, 1=Board named as party but not funder, 2=Funder | Contracts | |  |
|  |  | Quality of regional behavioral health board relationship with child welfare | 0=No relationship, 1=Negative, 2=Neutral, 3=Positive | Interviews | |  |
|  | Regional coordination | Regional behavioral health board member(s) part of county START steering committee | 0=No, 1=Yes | Interviews | |  |
|  |  | Other formal mechanisms in place within the county for facilitating cross-sector collaboration (e.g., coalition, drug court), not specific to regional behavioral health board | 0=No, 1=Yes | Interviews | |  |
|  | Brokering | Regional behavioral health board helped connect child welfare agencies with behavioral health providers for START | 0=No, 1=Yes | Interviews | |  |
|  | Resource support | Regional behavioral health board provided any funding for START program or START clients | 0=No, 1=Yes | Interviews | |  |
| **Contextual factors** | Community need – severity of opioid crisis | Nalaxone administrations (per 100,000 population) | 1 – 4^c^ | Ohio Department of Public Safety | |  |
|  |  | Unintentional drug overdose (OD) deaths (per 100,000 population) | 1 – 4^c^ | Ohio Department of Health | |  |
|  |  | Emergency department (ED) visits for suspected OD for individuals age 11+ years (per 10,000 ED visits) | 1 – 4^c^ | Ohio Department of Health Violence and Injury Prevention | |  |
|  |  | Neonatal abstinence syndrome (NAS) among resident live births (per 100 live births) | 1 – 4^c^ | Ohio Hospital Association | |  |
|  |  | Overall county opioid overdose severity (combination of all four measures above)^d^ | 1 – 4^c^ | See above | |  |
|  | Community need – child welfare (CW) | Children in CW custody (per 1000) | 1 – 4^c^ | Ohio JFS | |  |
|  |  | Total CW reports screened in | 1 – 4^c^ | Ohio JFS | |  |
|  |  | Total number of CW cases transferred to ongoing case plan / case management | 1 – 4^c^ | Ohio JFS | |  |
|  |  | Overall child welfare severity (combination of all 3 measures above)^e^ | 1 – 4^c^ | Ohio JFS | |  |
|  | Local behavioral health treatment availability | % Child welfare cases referred to behavioral health providers within county | 1=<50%, 2=50-74%, 3=>74% | FLW survey | |  |
|  |  | % Child welfare cases referred to behavioral health providers outside of the county | 1=<50%, 2=50-74%, 3=>74% | FLW survey | |  |
|  | Prior history of collaboration | Child welfare agency had formal relationships with substance abuse treatment providers in place prior to START | 0=No, 1=Some, 2=All partners | Interviews | |  |
|  |  | Child welfare agency had formal relationships with other stakeholders in the community (e.g., courts) prior to START | 0=None, 1=Any | Interviews | |  |
|  | County sociodemographics | Rurality | 1=Rural, 2=Appalachian, 3=Suburban, 4=Urban | Ohio Department of Health & Ohio Department of Mental Health and Addiction Services | |  |
|  |  | % population enrolled in Medicaid | 1 – 4^c^ | Census Bureau | |  |
|  |  | County poverty rate | 1 – 4^c^ | Census Bureau | |  |
|  |  | County size (population) | 1 – 4^c^ | Census Bureau | |  |
|  |  | % population non-Hispanic/Latino white | 1 – 4^c^ | Census Bureau | |  |
|  | Other community context | Regional behavioral health board serves >1 county | 0=No, 1=Yes | OACBHA | |  |
|  |  | Whether county served minimum clients needed for START certification (25 cases) | 0=No, 1=Yes | Needs Portal | |  |
| **Organizational factors** | Child welfare agency structure | Whether county Children’s Services Board is combined with or separate from Job & Family Services | 1=Separate, 2=Combined, 3=Hybrid | Ohio JFS | |  |
|  |  | Whether county child welfare has an active behavioral health levy in place | 0=No, 1=Yes | Ohio JFS | |  |
|  | Implementation leadership | Leadership for START implementation (12-item Implementation Leadership Scale [ILS]) | 0=Below average, 1=Average, 2=Above average^b^ | FLW survey | |  |
|  |  | Proactive leadership, ILS subscale | 0=Below average, 1=Average, 2=Above average^b^ | FLW survey | |  |
|  |  | Knowledgeable leadership, ILS subscale | 0=Below average, 1=Average, 2=Above average^b^ | FLW survey | |  |
|  |  | Supportive leadership, ILS subscale | 0=Below average, 1=Average, 2=Above average^b^ | FLW survey | |  |
|  |  | Perseverant leadership, ILS subscale | 0=Below average, 1=Average, 2=Above average^b^ | FLW survey | |  |
|  | Implementation climate | Extent to which START implementation is prioritized and valued (18-item Implementation Climate Scale [ICS]) | 0=Below average, 1=Average, 2=Above average^b^ | FLW survey | |  |
|  |  | Extent to which START implementation is expected, ICS subscale | 0=Below average, 1=Average, 2=Above average^b^ | FLW survey | |  |
|  |  | Extent to which START implementation is supported, ICS subscale | 0=Below average, 1=Average, 2=Above average^b^ | FLW survey | |  |
|  |  | Extent to which START implementation is rewarded, ICS subscale | 0=Below average, 1=Average, 2=Above average^b^ | FLW survey | |  |
|  | Implementation challenges | Challenges with implementing the family peer mentor role | 0=No challenges, 1=Challenge with recruitment/retention or conflict between family peer mentor and child welfare, 2=Both | Interviews | |  |

FLW = Frontline worker; OACBHA = Ohio Association of County Behavioral Health Authorities; JFS = Department of Job & Family Services

^a^ Wilder Collaboration Factor Inventory (WCFI) is a validated instrument that assesses six categories of factors shown to affect collaboration (Mattessich et al., 2001), including (1) the environment in which the collaboration is located, (2) partner characteristics, (3) collaboration process and structure, (4) cross-agency communication, (5) clarity of collaboration purpose, and (6) availability of resources to support collaboration endeavors.

^b^ We constructed two versions of these variables: (a) Values based on percentile within the state; Below average=<33^th^ percentile; Average=33-66^th^ percentile; Above average=>67^th^ percentile; and (b) Values based on responses on the 1-5 Likert scale (Below average=<2.5, Average=2.5-4.5, Above average=>4.5).

^c^ Values represent county quartile within the state; 1=<25^th^ percentile, 2=25-49^th^ percentile, 3= 50-74^th^ percentile, 4= >75^th^ percentile\

^d^ County opioid overdose severity was calculated by first generating quartile values (0-3) for the following four measures: naloxone administration rates, age-adjusted unintentional drug overdose deaths per 100,000, emergency department visits for suspected drug overdose among Ohio residents aged 11+ years per 10,000 ED visits, and neonatal abstinence rate among Ohio resident live births. Quartile values were summed to calculate overall severity, and then calibrated as mild (0-2), moderate (3-5), severe (6-8), and critical (9-12).

e County child welfare severity was calculated by first generating quartile values (0-3) for the following three measures: children in child welfare custody per 1000, total child welfare reports screened in, and total number of child welfare cases transferred to open cases/ongoing case management. Quartile values were summed to calculate overall severity and then calibrated as mild (0-1), moderate (2-4), severe, (5-6), and critical (7-9).

**Appendix 4. Configurational comparative methods**

CCMs represent formal, mathematical approaches to conducting cross-case analyses that are based in Boolean algebra and set theory (52); a critical analytic objective is to identify “difference-making” combinations of conditions that uniquely distinguish one group of cases from another. An advantage of these methods over correlation-based techniques for studying implementation of complex interventions is that analyses allow for both causal complexity (when several conditions must jointly appear for an outcome to occur) and equifinality (multiple pathways to an outcome) (53). In CCMs, positive models (solutions explaining when an outcome is present) are run separately from negative models (solutions explaining when an outcome is absent).

Currently, the two most prominent types of CCMs are qualitative comparative analysis (QCA) and coincidence analysis (CNA). The two methods are both based in Boolean algebra and set theory, but differ in the algorithms used to identify necessary and sufficient conditions (54). In the current study, we chose to use CNA because it does not require the use of counterfactuals, i.e., making assumptions about configurations not observed in our data, and because CNA is the only CCM with a systematic routine to assist with initial factor selection.
